# Marginal and Internal Adaptation of Implant‐Supported Three‐Unit Metal Frameworks Fabricated by the Conventional, Semi‐Digital, and Fully Digital Techniques Before and After Porcelain Application

**DOI:** 10.1002/cre2.70173

**Published:** 2025-07-09

**Authors:** Mansour Karimi, Hamid Neshandar Asli, Yeganeh Hamrah, Mohammad Ebrahim Ghaffari, Mehran Falahchai

**Affiliations:** ^1^ School of Dentistry Guilan University of Medical Sciences Rasht Iran; ^2^ Department of Prosthodontics, Dental Sciences Research Center, School of Dentistry Guilan University of Medical Sciences Rasht Iran; ^3^ Department of Epidemiology and Biostatistics, Faculty of Health Qom University of Medical Sciences Qom Iran

**Keywords:** CAD‐CAM, dental internal adaptation, dental marginal adaptation, implant‐supported dental prosthesis

## Abstract

**Objectives:**

Only a small number of studies conducted on implant‐supported fixed multi‐unit restorations have evaluated the semi‐digital fabrication techniques. This study aimed to assess the marginal and internal adaptation of implant‐supported three‐unit metal frameworks fabricated by the conventional, semi‐digital, and fully digital techniques before and after porcelain application.

**Material and Methods:**

In this in vitro study, 120 three‐unit metal frameworks were fabricated by five different techniques (*n* = 20): fabrication of metal frameworks from hard metal by the milling technique, direct 3D‐printing of metal, milling of resin pattern and subsequent casting, 3D‐printing of resin pattern and subsequent casting, and conventional waxing and subsequent casting. The marginal and internal adaptation of the frameworks was evaluated before and after porcelain application by using the silicone replica technique. Data were analyzed using ANOVA followed by pairwise comparisons with the Games‐Howell and paired samples tests (α = 0.05).

**Results:**

Before porcelain application, resin pattern milling, and subsequent casting resulted in the smallest marginal gap, while hard metal milling yielded the largest marginal gap. The fully digital techniques yielded the largest cuspal and fossa gaps, while the conventional method yielded the largest axial gap. After porcelain application, metal 3D‐printing and conventional casting resulted in comparable (*p* = 0.109) marginal gaps, smaller than hard metal milling (*p* < 0.001). The conventional casting method yielded the smallest cuspal and fossa gaps (*p* < 0.001). Porcelain application significantly increased the gap size at all measurement points (*p* < 0.001).

**Conclusion:**

The fabrication technique significantly affected the marginal and internal adaptation of implant‐supported three‐unit metal frameworks both before and after porcelain application.

## 1 Introduction

Precise seating of restorations is imperative to optimally meet the biological, physical, and esthetic requirements (Addugala et al. 2022). Marginal adaptation is a key factor in the assessment of the prognosis of fixed partial dentures and can affect their clinical success (Bae et al. 2020). Several studies have shown that poor adaptation of implant restorations can lead to mechanical failures such as gold screw loosening or fracture, framework fracture, or veneering fracture (Alkanani and Alnuwaiser 2022; Jemt et al. 1992). Optimal marginal adaptation preserves periodontal health and minimizes cement washout, and a proper internal adaptation creates a perfectly uniform cement space that does not interfere with the retentive form or support of restoration during the cementation process (Falahchai et al. 2022; Sadighpour et al. 2021). According to the literature, the acceptable marginal gap threshold ranges from 50 to 300 µm (Moldovan et al. 2006; Ucar et al. 2009). McLean and von Fraunhofer (1971) stated that marginal gaps up to 120 µm are clinically acceptable, and the greatest consensus exists on this threshold.

Metal‐ceramic systems were introduced for dental restorations, aiming to combine the benefits of metal alloys, such as high tensile and compressive strength and resistance to wear and corrosion, with the excellent esthetic appearance of ceramics (Dawod et al. 2023). Metal frameworks can be fabricated by different methods, such as the conventional casting or the computer‐aided design/computer‐aided manufacturing (CAD/CAM) fabrication technique (Nakhaei et al. 2023). The lost‐wax or the conventional technique has several steps with high technical sensitivity, and deformation of the wax pattern, casting defects, and cooling shrinkage may lead to metal framework misfit (Yang et al. 2022). The CAD/CAM manufacturing technology may be divided into the subtractive and additive techniques. The subtractive technique is based on milling of a block to fabricate an object (Falahchai et al. 2022). Initially, frameworks used to be fabricated from cobalt‐chromium (Co‐Cr) alloy with empty spaces in the fully sintered phase (Önöral et al. 2018). However, material waste is the main drawback of these prefabricated blocks (Kayikci and Ates 2022). Also, drills used in this technique have a short lifespan (Kayikci and Ates 2022). The selective laser melting is a well‐known additive technique in which metal powder particles melted by a heat source such as laser or electron beam fabricate a three‐dimensional (3D) object layer by layer according to a CAD file (Al‐Saleh et al. 2022). The material waste is lower in this technique (Pasha et al. 2023). Overall, based on the level of digital integration, metal framework fabrication techniques can be broadly categorized into three groups: conventional (fully manual), fully digital (direct digital fabrication of the final framework), and semi‐digital (digital pattern fabrication followed by conventional casting).

It has been stated that thermal changes during the sintering and cooling processes can cause dimensional changes. The causes of this distortion, which can alter the marginal adaptation, include differences in the coefficients of thermal expansion of metal and ceramic, type of metal alloy, metal defects, and finish line design (Yildirim 2020). Although some studies have previously evaluated the marginal and internal adaptation of implant‐supported frameworks fabricated by different methods (Nakhaei et al. 2023; Kayikci and Ates 2022; Yildirim 2020; Pan et al. 2021; Pera et al. 2023; Cesmeci et al. 2023; Shah et al. 2020; Rismanchian et al. 2022; Usta Kutlu and Hayran 2024), the majority of them compared the conventional casting and fully digital (Nakhaei et al. 2023; Pan et al. 2021; Pera et al. 2023; Cesmeci et al. 2023; Shah et al. 2020) or different digital methods (Kayikci and Ates 2022; Yildirim 2020; Rismanchian et al. 2022) with each other, out of which only a small number focused on implant‐supported fixed multi‐unit restorations (Kayikci and Ates 2022; Moris et al. 2018), and less attention has been paid to semi‐digital fabrication workflows (Giti and Farrahi 2024). Moreover, studies regarding the effect of porcelain application and related firing on implant‐supported multi‐unit metal‐ceramic restorations fabricated by different techniques are limited in number (Biçer and Mutluay Ünal 2023). Therefore, the purpose of this study was to compare the marginal and internal adaptation of implant‐supported three‐unit metal frameworks fabricated by the conventional, semi‐digital, and fully digital workflows before and after porcelain application. The null hypothesis of the study was that the framework fabrication method and porcelain firing would have no significant effect on the marginal and internal adaptation of implant‐supported three‐unit restorations.

## 2 Materials and Methods

This in vitro study used a custom‐made acrylic resin master model simulating a partially edentulous segment with two implant analogs embedded at the sites of the second premolar and second molar; 120 three‐unit metal frameworks were fabricated on it using five different fabrication methods (*n* = 20): (i) fabrication of framework from hard metal by the milling technique, (ii) fabrication of framework by direct 3D‐printing of metal, (iii) fabrication of resin pattern by the milling technique and subsequent casting, (IV) fabrication of resin pattern by 3D‐printing and subsequent casting, and (V) conventional fabrication of the wax pattern and subsequent casting.

Considering the measurement of marginal adaptation in five groups, the sample size was calculated using the formula for the comparison of the mean and standard deviation values of the five groups according to the performance curve. The sample size was calculated to be 20 in each group according to a previous study (Örtorp et al. 2011) and assuming 0.95 statistical power, 0.05 error rate, and a standard deviation of 134.

A custom acrylic resin block was fabricated using a perforated metal mold (30 mm length × 10 mm width × 20 mm height) filled with auto‐polymerizing acrylic resin (Acropars; Marlic Medical Instruments Co., Tehran, Iran). To simulate a partially edentulous segment suitable for a three‐unit prosthesis, two cavities were prepared in the buccolingual center of the block using a round bur, ensuring adequate spacing between the implant sites. Two regular‐diameter implant analogs (SASA5807; Dio Implant, Busan, Republic of Korea) were then positioned into the prepared cavities. To ensure proper alignment, impression copings were attached to the analogs, and the assembly was mounted using the vertical arm of a dental surveyor, aligning the analogs perpendicular to the horizontal plane and parallel to each other. While still connected to the surveyor, the remaining space around the analogs was filled with auto‐polymerizing resin to stabilize their position within the block (Ghodsi et al. 2019).

**Figure 1:**
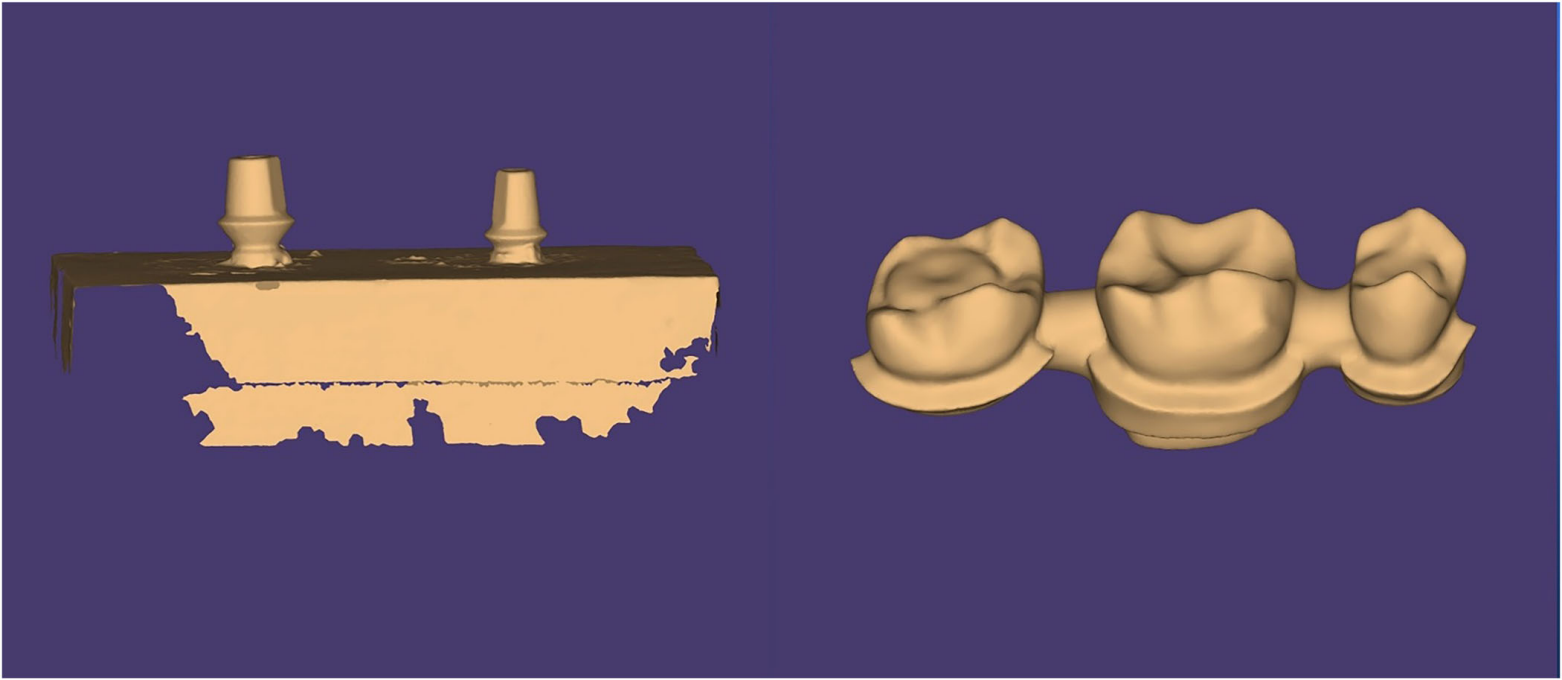
Scanned master model and a representative designed framework in the software environment.

The prepared samples were scanned using a laboratory scanner (Ceramill Map 600; Amann Girrbach AG, Koblach, Austria), and the frameworks were designed using CAD software (Ceramill Mind; Amann Girrbach AG, Koblach, Austria). The cement space was considered to be 40 µm and extended to 0.5 mm to the margins (Ghodsi et al. 2019). The framework thickness was 0.5 mm, and the connector area was considered to be 9 mm^2^. The pontic area was also designed (Figure 1).

**Figure 2:**
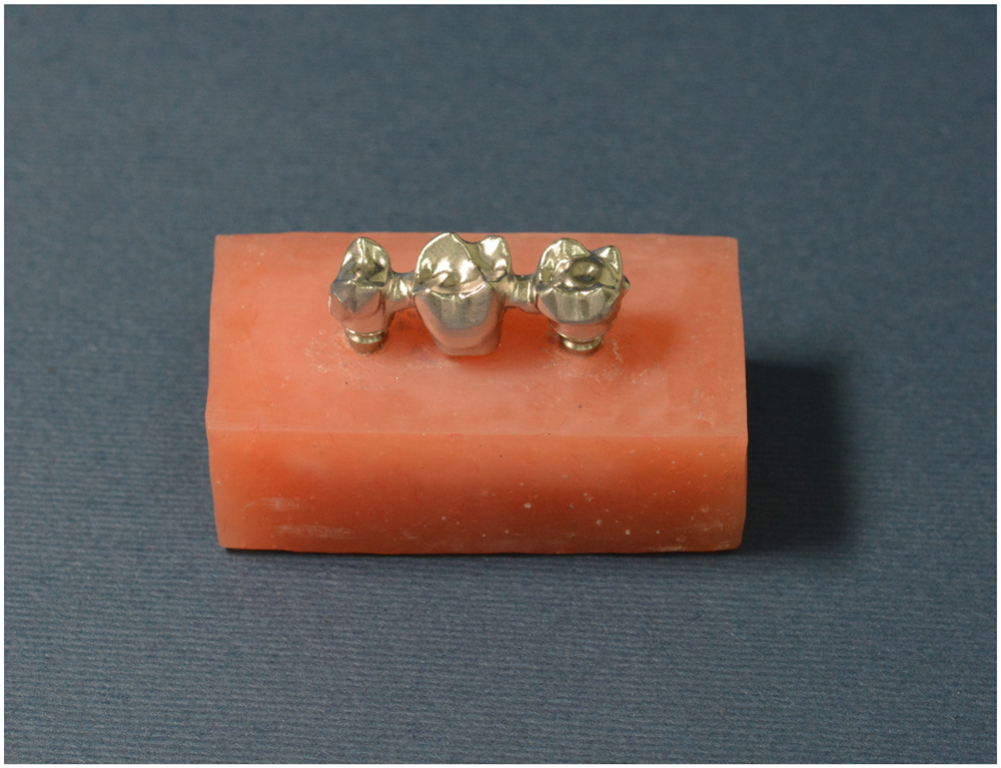
A representative metal framework fabricated by the hard metal milling technique.

In the first technique, the frameworks were fabricated according to the CAD design by milling of the sintered Co‐Cr metal blocks (Ceramill CoCr; Amann Girrbach AG, Koblach, Austria) using a 5‐axis milling machine (X‐400; Arum Dentistry, Daejeon, Republic of Korea) (Figure 2).

In the second technique, metal frameworks were fabricated according to the CAD design by the selective laser melting technology using the Co‐Cr alloy powder (Starbond Easy Powder 30; GmbH, Mainz, Germany) and a 3D printer (Riton Ti150). The 3D coping was positioned vertically. The support was subsequently designed and connected to the coronal part of the copings in the software. Each fusion layer had a 20 µm thickness, and the building platform moved down during the process by deposition of a new powder layer. This process was repeated until complete fabrication and sintering of the coping.

In the third technique, the wax patterns (Ceramill Wax; Amann Girrbach AG, Koblach, Austria) were milled in a milling machine (5X‐400; Arum Dentistry, Daejeon, Republic of Korea) and invested in phosphate‐bonded investment (Z4‐C&B investment; N&V, Schelle, Belgium). After burn‐out of the wax patterns at 950°C for 40 min, the mold was filled with Co‐Cr metal alloy (Flexicast; American Dent‐All Inc, CA, USA) and cast in a casting machine (Ducatron; KFP Dental Co, Tehran, Iran).

In the fourth technique, resin patterns were printed by a 3D printer (Alexandria, Australia Freeform PRO2; Asiga) with the digital light processing technology using the previous STL design file. Subsequent investing and casting were performed as described for group 3.

In the fifth technique, a silicone index (Speedex; Coltene, Altstatten, Switzerland) was obtained from a metal coping fabricated in group 1. After the application of two layers of die spacer (Pico‐fit; Renfert GmbH), melted wax (Blue Inlay Casting wax; Ramin Moom, Tehran, Iran) was added to each abutment through a hole created in the silicone occlusal index to 0.5 mm to the margin with a total thickness of 40 µm (Giti and Farrahi 2024). After removal and final adjustments of the wax patterns, the samples were evaluated to ensure the absence of visible gaps between the pattern and the die margin. In the next step, the wax patterns formed on the abutments were connected to each other with a wax sprue. Subsequently, the investment and casting processes were performed as explained for Group 3. All fabrication steps in each group were performed by the same experienced technician with the same parameters.

The fabricated frameworks by each technique were seated on the master model for assessment of their marginal and internal adaptation. The silicone replica technique was used for this purpose. Light body silicone impression material (Panasil; Kettenbach, CA, USA) was poured into each framework, and it was placed on the master model. After polymerization of the silicone material, the framework was carefully separated from the die, with the silicone material remaining in it. Next, medium‐body silicone material was injected into the framework, and light‐body silicone was injected over it by an injection gun to stabilize the light‐body silicone. Subsequently, the silicone replica of each abutment was sectioned mesiodistally and buccolingually with a razor blade. To assess the internal discrepancy, the vertical distance between the internal surface of the crown and the external surface of the preparation was measured at the central points of the axial, cuspal, and fossa areas. For each abutment, the marginal, axial, cuspal, and fossa gaps were measured at the buccal, lingual, mesial, and distal of each sample. In other words, 16 measurements were made for each abutment, and 32 measurements were made for each sample (including two abutments). The measurements were made under a video measuring machine (Easson Optoelectronics, Suzhou, China) at x132.8 magnification by a blinded examiner.

To assess the effect of porcelain application on adaptation of the samples, only the frameworks in groups 1, 2, and 5 underwent porcelain (Vintage Pro Starter Set A2/A3; Shofu Dental Co., CA, USA) application since the same alloy was used in the three conventional casting groups. After airborne particle abrasion and subsequent cleaning of the frameworks in an ultrasonic bath as instructed by the manufacturer, porcelain was applied and fired in a dental ceramic furnace (AT300: KFP Dental Co., Tehran, Iran). To prevent possible adaptation measurement errors, a 0.5‐mm area at the cervical margin remained without a veneering ceramic. Uniform ceramic thickness (0.5–1 mm) was ensured by using silicone indexes obtained through a mock‐up. The porcelain firing process included initial oxidation, opaque firing, dentin/body firing, enamel/incisal firing, laboratory correction firing, clinical correction firing, and glaze firing (Giti and Farrahi 2024). After porcelain firing, all measurements were repeated at the predetermined points using the silicone replica technique.

The Shapiro–Wilk test was used to analyze the normality of data distribution, while the homogeneity of the variances was analyzed using the Levene test. ANOVA, followed by the Games‐Howell test, was used to compare marginal adaptation of the study groups, while paired samples test was applied to compare gaps before and after porcelain application. All statistical analyses were conducted using SPSS version 28 (IBM SPSS Statistics, v26; IBM Corp, NY, USA) at a 0.05 level of significance.

## 3 Results

Before porcelain application, a significant difference was found among marginal, axial, cuspal, and central fossa gaps in all fabrication methods (*p* < 0.001), and the cuspal gap was the largest in all groups. The axial gap was the smallest gap in the hard metal milling, metal 3D‐printing, and resin pattern 3D‐printing groups, while the smallest gap was the marginal gap in the resin pattern milling and conventional groups. All pairwise comparisons were significant in all methods except for the metal 3D‐printing (*p* < 0.001). In the metal 3D‐printing, no significant difference existed between the marginal and axial gaps (*p* = 0.262), while they were significantly different in other groups (*p* < 0.001, Table 1).

**Table 1:** Comparison of the mean gap size at different measurement points of three‐unit metal frameworks fabricated by different methods before porcelain application.

Gap	Fabrication method					*p* value (statistic)
	Hard metal milling Mean ± SD	Metal 3D‐printing Mean ± SD	Resin pattern milling Mean ± SD	Resin pattern 3D‐printing Mean ± SD	Conventional Mean ± SD	
**Marginal gap**	83.20 ± 80.11^Aa^	70.63 ± 49.12^Ba^	59.58 ± 07.14^Ca^	69.71 ± 23.10^Ba^	65.80 ± 57.20^Ba^	< 0.001 (58.90)
**Axial gap**	66.57 ± 28.25^ABb^	67.15 ± 57.20^ABa^	66.94 ± 40.7^Bb^	63.47 ± 06.11^Ab^	85.03 ± 67.8^Cb^	< 0.001 (45.30)
**Cuspal gap**	120.09 ± 25.71^Ac^	106.34 ± 37.32^Bb^	91.45 ± 74.18^Cc^	99.61 ± 85.23^BDc^	94.12 ± 36.25^CDc^	< 0.001 (45.30)
**Fossa gap**	172.41 ± 58.30^Ad^	168.61 ± 38.58^Ac^	127.68 ± 17.15^Bd^	121.59 ± 04.12^Cd^	105.67 ± 62.40^Dd^	< 0.001 (45.30)
***p* value (statistic)**	< 0.001 (592.91)	< 0.001 (281.66)	< 0.001 (723.40)	< 0.001 (498.21)	< 0.001 (65.02)	

*Note:* ANOVA with Games‐Howell test. Similar uppercase letters in the same row and similar lowercase letters in the same column indicate the absence of a significant difference (*p* > 0.05).

Different fabrication techniques had a significant difference with each other at all gap measurement points before porcelain application (*p* < 0.001). The marginal gap in the hard metal milling group was significantly larger than that in all other groups (*p* < 0.001). The marginal gap in the resin pattern milling group was significantly smaller than that in all other groups (*p* < 0.05). The difference between other groups was not statistically significant (*p* > 0.05). The axial gap in the conventional method was significantly larger than that in other methods (*p* < 0.001). The axial gap in the resin pattern 3D‐printing group was significantly smaller than that in the resin pattern milling group (*p* = 0.010). No significant difference existed between other methods in this regard (*p* > 0.05). The cuspal gap in the hard metal milling method was significantly larger than that in other methods (*p* < 0.001). The fossa gap was the largest in the hard metal milling and metal 3D‐printing groups and was significantly larger than that in other groups (*p* < 0.001). However, the conventional method showed a significantly smaller fossa gap than other methods (*p* < 0.001, Table 1).

Similar uppercase letters in the same row and similar lowercase letters in the same column indicate the absence of a significant difference (*p* > 0.05).

**Figure 3:**
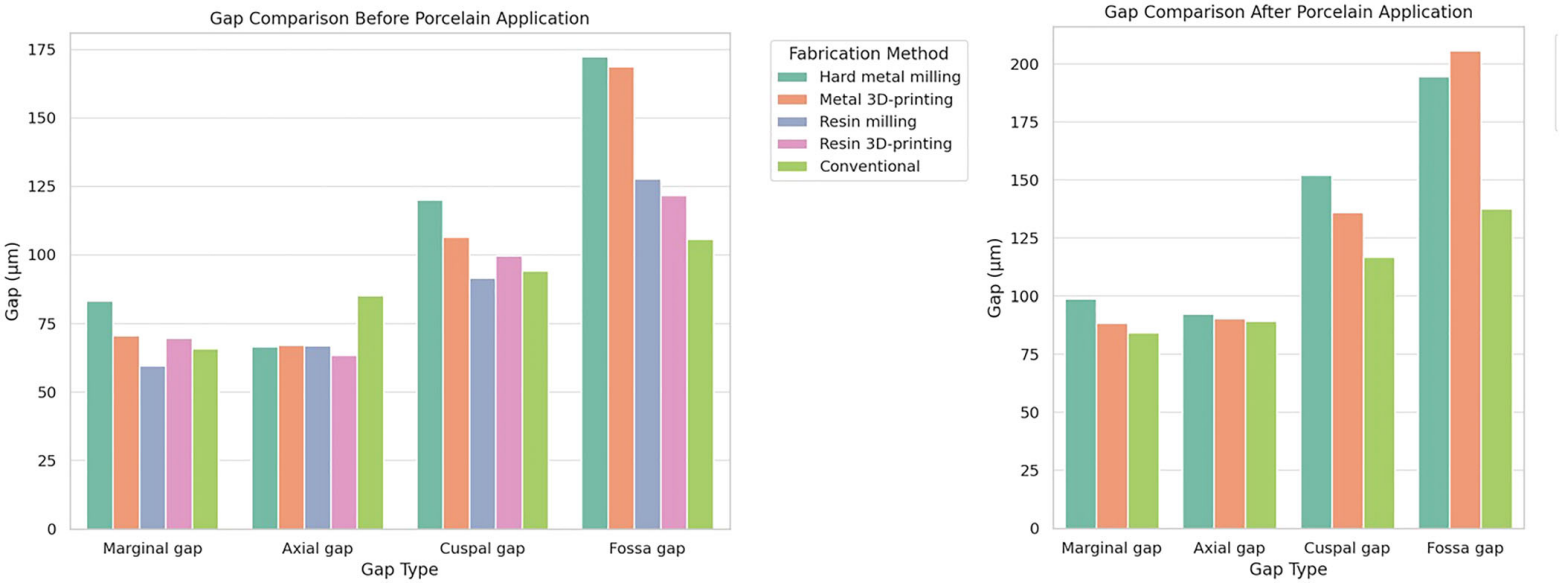
Comparison of marginal, axial, cuspal, and fossa gaps among different fabrication techniques before and after porcelain application.

Table 2 presents the results after porcelain application. In all fabrication methods, a significant difference was found among marginal, axial, cuspal, and fossa gaps (*p* < 0.001). All pairwise comparisons were significant (*p* < 0.05). The smallest gap was the marginal gap in the metal 3D‐printing and the conventional methods, and the axial gap in the hard metal milling group. The largest gap was recorded at the central fossa in all groups. Comparison of the fabrication methods with each other revealed significant differences in marginal, cuspal, and fossa gaps (*p* < 0.001). The largest marginal and cuspal gaps were recorded in the hard metal milling group, while the largest fossa gap was noted in the metal 3D‐printing group. The metal 3D‐printing and conventional groups had no significant difference regarding the marginal gap (*p* = 0.109). Nonetheless, the conventional method yielded a smaller cuspal and fossa gap than the metal 3D‐printing group (*p* < 0.001). No significant difference was found among the fabrication groups in axial gap (*p* = 0.226). A visual comparison of gap values across fabrication techniques is presented in Figure 3.

**Table 2:** Comparison of the mean gap size at different measurement points of three‐unit metal frameworks fabricated by different methods after porcelain application.

	Marginal gap	Axial gap	Cuspal gap	Fossa gap	*p* value (statistic)
**Hard metal**	98.8 ± 15.39^Aa^	92.36 ± 26.71^B^	152.11 ± 24.73^Ca^	194.51 ± 30.79^Da^	< 0.001 (591.09)
**Metal 3D‐printing**	88.47 ± 23.11^Ab^	90.35 ± 24.56^B^	135.94 ± 34.89^Cb^	205.81 ± 39.90^Db^	< 0.001 (490.71)
**Conventional**	84.33 ± 20.39^Ab^	89.36 ± 13.12^B^	116.74 ± 22.85^Cc^	137.52 ± 30.85^Dc^	< 0.001 (574.62)
***p* value (statistic)**	< 0.001 (31.54)	0.226 (1.49)	< 0.001 (120.71)	< 0.001 (353.85)	

*Note:* Similar uppercase letters in the same row and similar lowercase letters in the same column indicate the absence of a significant difference (*p* > 0.05).

Paired samples test showed a significant difference in marginal, axial, cuspal, and fossa gaps before and after porcelain application in all fabrication methods (*p* < 0.001). In all methods, the mean gap at different measurement points increased following the porcelain application.

## 4 Discussion

The present study assessed the marginal and internal adaptation of implant‐supported three‐unit metal frameworks fabricated by the conventional, semi‐digital, and fully digital techniques before and after porcelain application. The results revealed significant differences among the groups. Thus, the null hypothesis of the study was rejected.

Different methods are used for assessment of adaptation, such as the cross‐sectional and silicone replica techniques, direct microscopic observation, and micro‐computed tomography (Kim et al. 2018; Yildirim and Paken 2019; Nawafleh et al. 2013). In the present study, the silicone replica technique was employed for this purpose since it allows assessment of both marginal and internal gaps (Ghavami‐Lahiji et al. 2023). The silicone replica technique is noninvasive and cost‐effective and allows precise and reproducible measurement of gap at different points. It has been used in many previous studies as well (Falahchai et al. 2022; Önöral et al. 2018; Nawafleh et al. 2013; Kim et al. 2014; D.‐Y. Kim, J.‐H. Kim, et al. 2017; Nesse et al. 2015; Ardekani et al. 2024). This study required reproducible measurements.

Marginal adaptation is the most important criterion considered by dental clinicians for the acceptability of cast restorations (Huang et al. 2015). The majority of researchers use the McLean and Fraunhofer (1971) threshold for the maximum clinically acceptable marginal gap. They evaluated over 1000 restorations and concluded that marginal gaps < 120 µm are clinically acceptable. The marginal gaps in all groups of the present study were within the clinically acceptable range (< 120 µm) both before and after porcelain application. Although comparison of gap size among different studies is not accurately feasible, differences may be related to conduction or no conduction of cementation and thermocycling and differences in adaptation assessment methods, accuracy of scanners, types of 3D‐printers and milling machines, and materials used (Bae et al. 2020; Thakur et al. 2023).

In the present study, the fully digital hard metal milling technique yielded the largest marginal gap, and the semi‐digital resin pattern milling and subsequent casting yielded the smallest marginal gap before porcelain application. No significant difference existed between other groups. After porcelain application, the hard metal milling technique yielded the largest marginal gap, and no significant difference was found between the metal 3D‐printing and conventional casting techniques. The milling process can result in higher residual stress in the copings compared with the metal 3D‐printing and conventional casting (Giti and Farrahi 2024). Moreover, achieving 100% material density is one advantage of the metal 3D‐printing method (Thakur et al. 2023). Improved (decreased) marginal gap in the metal 3D‐printing compared with the hard metal milling technique in the current study was in line with previous findings (Kayikci and Ates 2022; Usta Kutlu and Hayran 2024; Thakur et al. 2023; Akçin et al. 2018). Akçin et al. (2018) found no significant difference in adaptation of implant‐supported three‐unit frameworks fabricated by metal printing and conventional casting techniques, and metal milling resulted in poorer adaptation. The majority of studies that reported a different result used nickel chromium alloy (Kim et al. 2013; Pompa et al. 2015). Giti and Farrahi (2024) evaluated the effect of resin pattern fabrication methods and showed that resin pattern 3D‐printing yielded a smaller marginal gap than resin pattern milling and conventional wax‐up methods. This difference can be attributed to differences in methodology, such as the adopted technique for gap measurement. Higher adaptation in the resin pattern milling and subsequent casting technique compared with the hard metal milling technique in the current study was also in agreement with previous findings (Ghodsi et al. 2019) and may be attributed to greater wear and degradation of burs in hard metal milling.

Poor internal adaptation would result in a thick, nonuniform cement layer and subsequently increased shear stresses at the restoration‐abutment interface, which can exceed the bond strength and lead to debonding (Moldovan et al. 2011). In all fabrication methods in the present study, the cuspal and fossa gaps were larger than the axial and marginal gaps, which was in agreement with previous findings (Örtorp et al. 2011; Akçin et al. 2018). The cuspal and fossa gaps in the hard metal milling and metal 3D‐printing methods were larger than the corresponding values in the conventional method, both before and after porcelain application. The milling process has some limitations. For instance, details smaller than the diameter of the milling burs cannot be accurately fabricated. The adaptation of internal restoration surface also depends on the size of the smallest usable bur. If the bur diameter is larger than some parts of the restoration, the CAD/CAM system would face a difficult situation. In case of milling, the internal adaptation would decrease, and in case of no milling, the marginal adaptation would decrease (Tinschert et al. 2004; Reich et al. 2005). As reported in some studies, an inverse correlation exists between the internal adaptation and marginal adaptation in CAD/CAM systems (Kokubo et al. 2005; Han et al. 2011; Rocha et al. 2007; Aboushelib et al. 2012). Similarly, in the present study, although the hard metal milling technique yielded a larger marginal gap than other methods, it was still acceptable (< 120 µm). Also, the majority of the milling tools are not capable of creating precisely sharp internal corners, leading to a premature internal contact and a subsequently open margin. To prevent it, either a spacer parameter should be selected for the CAD/CAM system or the crown has to be adjusted by a technician during the laboratory try‐in process. Both of these procedures can increase the internal gap (Reich et al. 2005). Another explanation for a larger internal gap in the digital methods is the overshoot phenomenon that simulates virtual points close to the margins (Reich et al. 2005). Also, scanning spray, which is used to decrease surface reflection during scanning, is another potential factor that can increase the internal gap in the digital techniques, since it forms a distinct layer on the die surface. These results were consistent with the findings of Kim, Choi, et al. (2017) who reported superior internal adaptation of the conventional casting method. Nonetheless, Sadr et al. (2022) demonstrated comparable internal adaptation of three fabrication methods of milling, metal 3D‐printing, and conventional casting in single‐unit restorations; however, after porcelain application, the CAD/CAM method yielded a superior internal adaptation.

Following porcelain application in the present study, the marginal, axial, cuspal, and fossa gaps significantly increased in all fabrication methods, which can be due to distortions caused by repeated porcelain firing cycles. Repeated porcelain firing cycles can lead to dissimilar expansion and shrinkage of metals and ceramics and cause framework distortion, which adversely affects the final adaptation (Giti and Farrahi 2024). Metal frameworks are subjected to repeated firing cycles at high temperatures during the porcelain application process (Akçin et al. 2018), which causes distortion in the metallurgical structure of the alloy and adversely affect the restoration adaptation. Type of alloy, degree of ceramic shrinkage, and difference in the coefficients of thermal expansion of ceramic and metal play a role in this process (Giti and Farrahi 2024). The effect of porcelain firing on adaptation of metal‐ceramic restorations has been previously investigated, mostly in single‐unit restorations (Quante et al. 2008; Kocaağaoğlu et al. 2017; Zeng et al. 2015; Lang et al. 1995). The majority of previous studies showed that porcelain firing increased the gap size (Yildirim 2020; Kaleli and Saraç 2017). Nonetheless, some studies did not find a significant change (Kocaağaoğlu et al. 2017; Giti et al. 2023), and some others even reported a reduction in gap size (Real‐Voltas et al. 2017). Variations in the reported results in this respect may be attributed to differences in methodologies, type of materials used, framework design, framework span and thickness, sintering method, and method of gap measurement (Giti and Farrahi 2024).

From a clinical perspective, the findings of this study provide practical guidance for selecting an appropriate fabrication workflow based on laboratory resources, case‐specific priorities, and esthetic or functional demands. When superior marginal fit is the main concern and full digital infrastructure is not available, semi‐digital techniques—particularly resin pattern milling followed by casting—may offer an effective and accessible solution. Conversely, fully digital workflows should be selected with awareness of their specific limitations: hard metal milling may compromise marginal adaptation due to mechanical constraints, whereas metal 3D‐printing, while achieving better marginal fit, may present internal discrepancies caused by sintering distortions. Therefore, clinicians and technicians should tailor their workflow choices accordingly and consider compensatory steps during design, try‐in, and cementation to optimize clinical outcomes.

In vitro design and absence of saliva, occlusal forces, cement, and thermal alterations were the main limitations of this study. The lack of occlusal load simulation and omission of actual cementation may limit the ability to fully replicate intraoral conditions, as functional stresses and the mechanical behavior of luting agents can influence marginal and internal adaptation. Moreover, not using a 3D measurement tool for adaptation assessment was another limitation. Future research should consider load cycling, thermomechanical aging, and cementation procedures to better mimic clinical scenarios. In vitro studies on long‐span restorations and clinical studies on this topic are recommended.

## 5 Conclusion

Considering the limitations of this in vitro study, it may be concluded that the internal and marginal adaptation of implant‐supported three‐unit metal frameworks is influenced by their fabrication method. Nonetheless, irrespective of the fabrication method, porcelain application increased the gap size at all measurement points. Before porcelain application, the semi‐digital resin pattern milling, followed by the casting method, yielded the smallest marginal gap, while the fully digital hard metal milling technique yielded the largest marginal gap. Moreover, the fully digital techniques yielded the largest cuspal and fossa gaps, and the conventional method resulted in the largest axial gap. After porcelain application, the metal 3D‐printing and the conventional casting methods yielded a comparable marginal gap, smaller than that in the hard metal milling group. The largest cuspal gap was recorded in the hard metal milling, and the largest fossa gap was found in the metal 3D‐printing group.

## Author Contributions

**Mansour Karimi:** conceptualization, methodology, writing – review and editing. **Hamid Neshandar Asli:** supervision, methodology. **Yeganeh Hamrah:** resources, investigation, writing – original draft. **Mohammad Ebrahim Ghaffari:** data curation, data analysis. **Mehran Falahchai:** conceptualization, funding acquisition, project administration, writing – review and editing.

## Ethics Statement

This is an in vitro study (ethics code: IR.GUMS.REC.1399.188).

## Conflicts of Interest

The authors declare no conflicts of interest.

## Data Availability

The data that support the findings of this study are available from the corresponding author upon reasonable request.
